# An unusual case of Epstein-Barr virus-positive large B-cell lymphoma lacking various B-cell markers

**DOI:** 10.1186/s13000-017-0606-7

**Published:** 2017-01-31

**Authors:** Shin-ichi Nakatsuka, Chikao Yutani, Masako Kurashige, Masaharu Kohara, Teruaki Nagano, Takayoshi Goto, Hiroyuki Takatsuka, Hidetaka Ifuku, Eiichi Morii

**Affiliations:** 1 Department of Pathology, Kansai Rosai Hospital, 3-1-69 Inabaso, 660-8511 Amagasaki, Hyogo Japan; 2 Department of Pathology, Amagasaki Central Hospital, 1-12-1 Shioe, 661-0976 Amagasaki, Hyogo Japan; 3 Department of Pathology, Osaka University Graduate School of Medicine, 2-2 Yamadaoka, 565-0871 Suita, Osaka Japan; 4 Department of Internal Medicine, Amagasaki Central Hospital, 1-12-1 Shioe, 661-0976 Amagasaki, Hyogo Japan

**Keywords:** Malignant lymphoma, Epstein-Barr virus, Lineage specific marker, Downregulation, Oct-2, BOB.1, Gene rearrangement study

## Abstract

**Backgroud:**

Epstein-Barr virus (EBV) is associated with B-cell lymphoma in various conditions, such as immunodeficiency and chronic inflammation. We report an unusual case of EBV-positive diffuse large B-cell lymphoma (DLBCL) lacking the expression of many B-cell markers.

**Case presentation:**

An 83-year-old man presented with a submandibular tumor. Histology of a lymph node biopsy specimen revealed diffuse proliferation of centroblast- or immunoblast-like lymphoid cells with plasmacytic differentiation. Scattered Hodgkin/Reed-Sternberg-like cells were also visible. A routine immunohistochemistry antibody panel revealed that the tumor cells were negative for B-cell and T-cell markers (i.e., CD3, CD19, CD20, CD38, CD45RO, CD79a, CD138, and Pax-5), but were positive for CD30 and MUM-1, not defining the lineage of tumor cells. The final diagnosis of EBV-positive DLBCL was confirmed based on the expression of B-cell-specific transcription factors (Oct-2 and BOB.1), PCR-based identification of monoclonal rearrangement of the immunoglobulin genes, and the presence of EBV-encoded small RNAs in the tumor cells (identified using in situ hybridization).

**Conclusion:**

The downregulation of broad band of B-cell markers in the present case with EBV-positive DLBCL posed a diagnostic dilemma, as the possible diagnoses included differentiation from anaplastic large cell lymphoma and CD20-negative B-cell lymphomas. Results of immunohistochemical panel including B-cell-specific transcription factors and gene rearrangement analyses critically support the correct diagnosis.

## Background

Epstein-Barr virus (EBV) is associated with the pathogenesis of several B-cell neoplasms, such as Burkitt lymphoma, plasmablastic lymphoma (PBL), primary effusion lymphoma (PEL), immunodeficiency-associated lymphoproliferative disorders, and some diffuse large B-cell lymphomas (DLBCL), which include lyphomatoid granulomatosis and DLBCL with chronic inflammation [1, 2]. Most EBV-positive B-cell lymphomas, except for Burkitt lymphoma, develop in cases with systemic immunodeficiency or long-standing chronic inflammation, although B-cell lymphoma can infrequently develop in the absence of immunodeficiency or inflammatory conditions. These cases typically involve elderly patients, and some authors have described them as “EBV-positive DLBCL of the elderly” [3]. Previous studies have revealed that 2–11% of DLBCL cases are positive for EBV [4], and these cases are diagnosed based on the following findings: 1) diffuse infiltration of large lymphoid cells, 2) detection of B-cell markers during immunohistochemistry testing (IHC), and 3) the detection of EBV-encoded small RNAs using in situ hybridization. In this report, we describe an unusual case of EBV-positive DLBCL in an immunocompetent patient, which was difficult to diagnose due to the absence of many B-cell markers during routine IHC. Our final diagnosis was critically supported by the IHC identification of B-cell-specific transcription factors (Oct-2 and BOB.1) and findings from our gene rearrangement testing.

## Case presentation

### Clinical summary

**Fig. 1:**
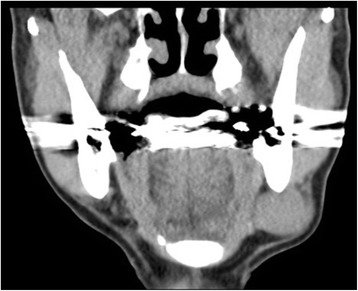
Computed tomography imaging of the tumor. A well-demarcated tumor is located in the left submandibular region (coronal plane)

An 83-year-old man noticed a left submandibular mass and visited our Department of Oral Surgery. He had no B symptoms. His family history was unremarkable, although he had a history of renal insufficiency. A physical examination revealed an elastic hard tumor (diameter: 4 cm) that was well-demarcated and had not adhered to the surrounding tissue. The covering skin was normal, and the patient reported not experiencing spontaneous pain or tenderness. Laboratory testing revealed slight anemia (red blood cell count: 385 × 10^4^/μL, hemoglobin levels: 11.7 g/dL) and leukopenia (white blood cell count: 3,900/μL), although his serum lactate dehydrogenase levels were not elevated (207 IU/L). Levels of soluble interleukin-2 receptor were markedly elevated (2,730 IU/mL), although we did not detect elevated titers of antibodies to human T-cell leukemia virus-1 or human immunodeficiency virus (HIV). Computed tomography and diffusion-weighted magnetic resonance imaging revealed a left submandibular tumorous lesion and multiple swollen lymph nodes (the left cervical and inguinal nodes) (Fig. 1). Magnetic resonance imaging also revealed multiple high-intensity areas in the vertebrae, bilateral ribs, and ilia, although there were no abnormalities in the mediastinum or abdomen. We obtained a biopsy specimen from the submandibular lesion, which supported our initial pathological diagnosis of anaplastic large cell lymphoma (ALCL), although a hematopathological consultant helped us make a final diagnosis of EBV-positive DLBCL. The patient underwent 2 courses of chemotherapy using the THP-COP regimen and achieved partial remission, although the tumor was ultimately resistant to the following therapies: molecular-targeted therapy using brentuximab vedotin, THP-COP (4’-O-tetrahydropyranyl adriamycin, cyclophosphamide, Oncovin®, prednisolone), and EPOCH (etoposide, prednisolone, Oncovin®, cyclophosphamide, hydroxydaunorubicin). The patient was alive with progressive disease at the 9-month follow-up.

### Histological evaluation

**Fig. 2:**
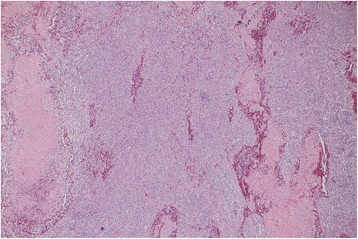
Low-magnification histological findings from the biopsy specimen. The lymph node architecture is effaced and occupied with diffuse infiltrative tumor cells. Foci of necrosis are visible (hematoxylin and eosin staining, original magnification: ×40)

**Fig. 3:**
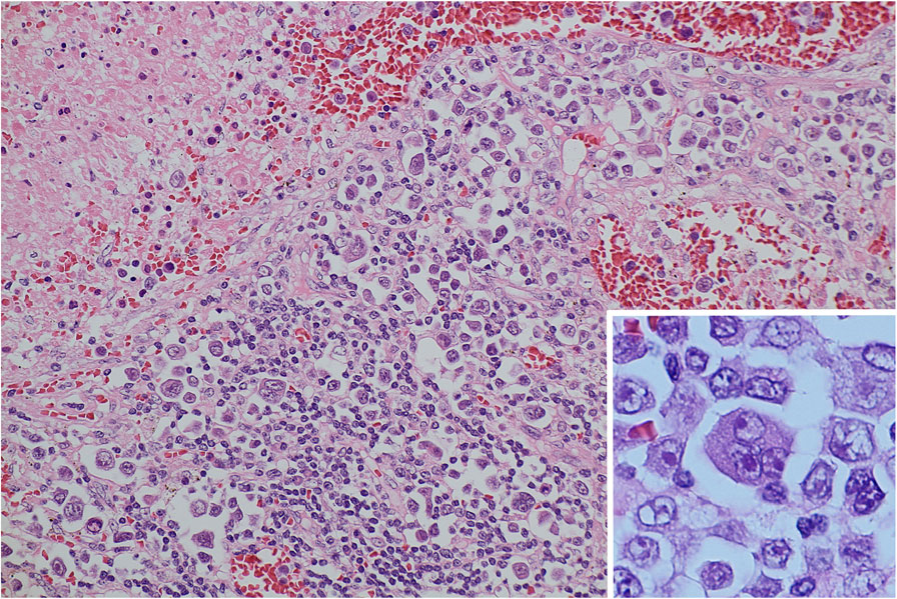
High-magnification histological findings from the biopsy specimen. Diffuse infiltration of large lymphoid cells that are similar to centroblasts or immunoblasts. Large multinuclear giant cells are scattered around the necrotic focus (hematoxylin and eosin staining, magnification: ×100). Hodgkin/Reed-Sternberg-like cells are also visible (inset)

Hematoxylin-eosin (HE) staining revealed that the lymph node architecture was effaced and diffusely occupied by infiltrative large lymphoid cells. Many foci of necrosis were visible, and the lesion was comprised of centroblast-like cells (a large vesicular nucleus and several conspicuous nucleoli), immunoblast-like cells (prominent central nucleoli), and plasmacytic cells (Figs. 2 and 3). There were scattered large multinuclear cells with prominent nucleoli, including large cells that were similar to Hodgkin/Reed-Sternberg cells (HRS-like cells) (Fig. 3).

**Fig. 4:**
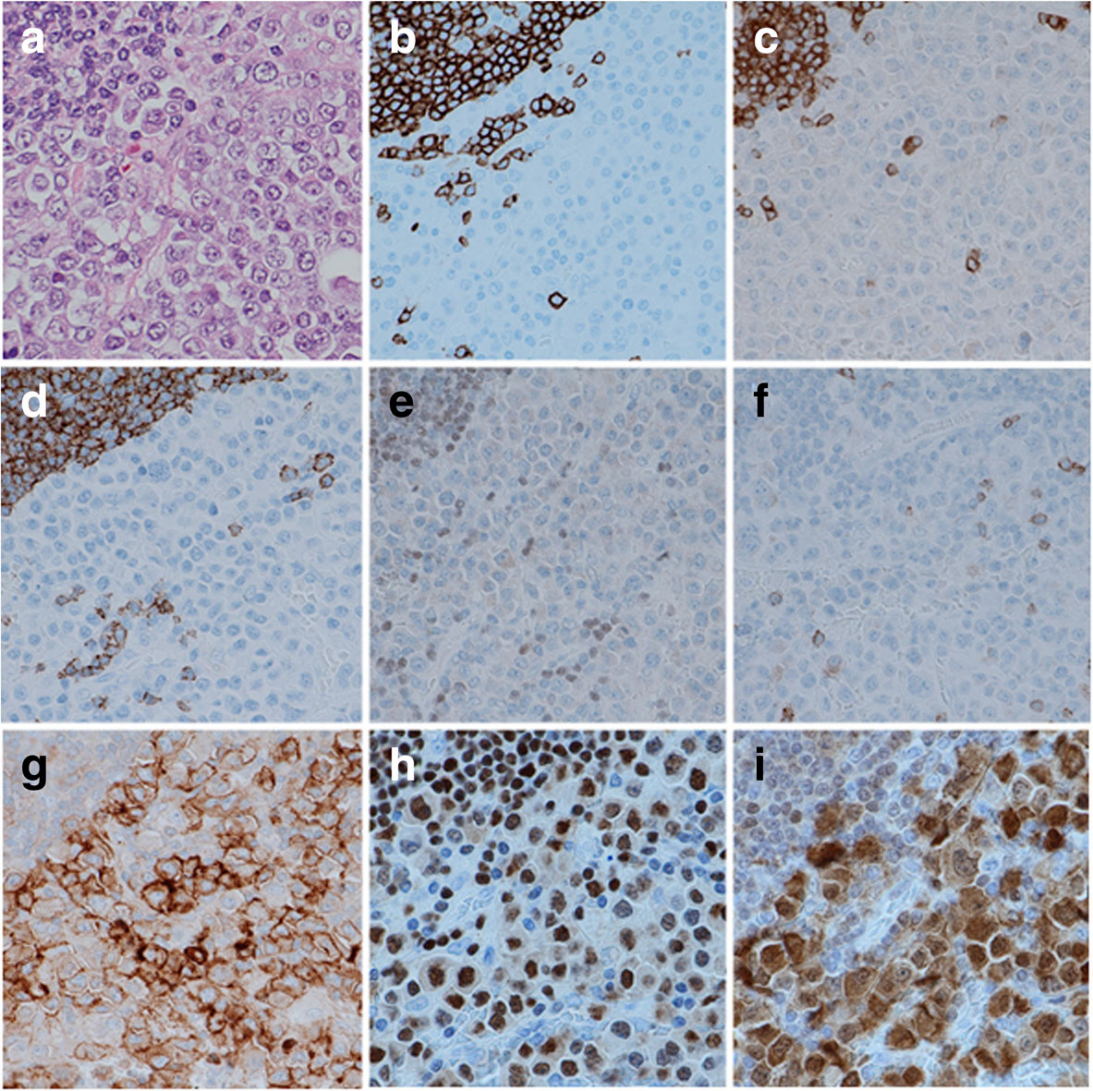
Immunohistochemistry findings from the biopsy specimen. Small lymphocytes seen in the upper left of photograph (**a**) are non-neoplastic B-cells in a lymph follicle (hematoxylin and eosin staining). The large neoplastic cells are negative for CD20 (**b**), CD79a (**c**), CD19 (**d**), Pax-5 (**e**), and CD3 (**f**), but are positive for CD30 (**g**), Oct-2 (**h**), and BOB.1 (**I**)

**Fig. 5:**
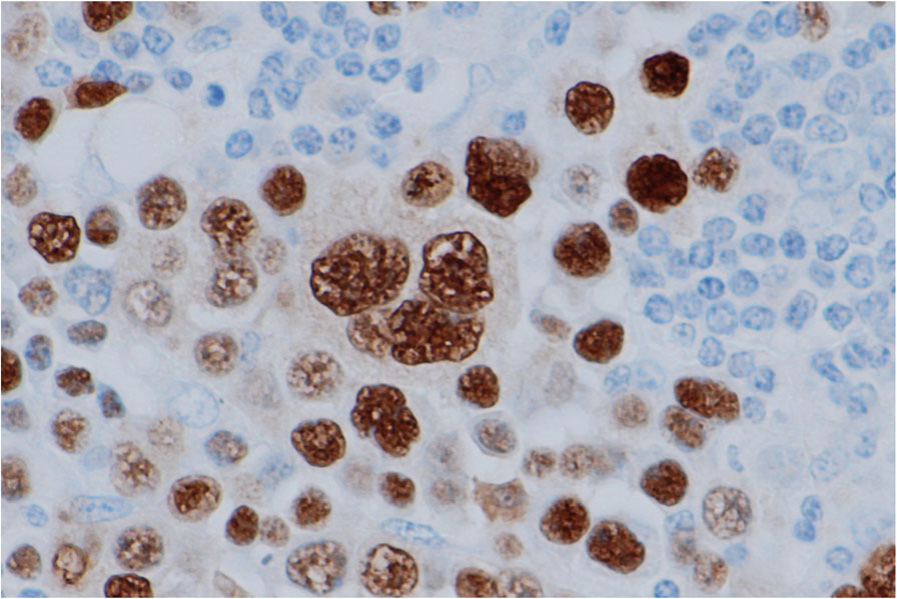
In situ hybridization findings. Most of the tumor cells, including the Hodgkin/Reed-Sternberg-like cells, exhibited positive signals for EBV-encoded small RNAs in the nucleus, which suggested a latent Epstein-Barr virus infection

The initial IHC revealed that the tumor cells were positive for CD30 and MUM-1, but negative for CD3, CD4, CD5, CD8, CD10, CD19, CD20, CD23, CD38, CD45, CD45RO, CD56, CD79a, CD138, Pax-5, immunoglobulin light chains (κ and λ), epithelial membrane antigen (EMA), anaplastic lymphoma kinase (ALK), Bcl-2, and Bcl-6 (Fig. 4b-g). These results could not define cell lineage and histological type of the tumor. Additional IHC revealed that the tumor cells were positive for B-cell-specific transcription factors (Oct-2 and BOB.1), which confirmed B-cell derivation of the tumor cells (Fig. 4h and i). In situ hybridization revealed clear positive signals for EBV-encoded small RNAs in the nuclei of the tumor cells (Fig. 5). The tumor cells were negative for LANA-1 (a product of human herpesvirus-8), and the Ki-67 labeling index was very high (approximately 80%).

### Clonality analysis

We performed gene rearrangement testing using the BIOMED-2 multiplex polymerase chain reaction-based method, which revealed clonal rearrangement of the immunoglobulin heavy chain and light chain genes. No clonal rearrangement was detected in the T-cell receptor genes.

## Discussion and conclusions

EBV-related lymphomas develop in various conditions, including systemic immunodeficiency and chronic inflammation [1, 2]. Most of these lymphomas are derived from B-cells, and EBV-positive B-cell lymphomas usually express several B-cell surface markers, although they occasionally lack one or more markers. However, few studies have evaluated the frequency of B-cell marker loss in EBV-positive B-cell lymphomas. One flow cytometry-based study revealed that 4 of 25 cases (16%) of posttransplant lymphoproliferative disorder exhibited almost complete loss of CD20 expression (all 4 CD20-negative cases were EBV-positive), compared to only 8 of 334 cases (2%) of *de novo* B-cell non-Hodgkin lymphoma [5]. Another study revealed a higher incidence of decreased CD19 expression in cases of posttransplant lymphoproliferative disorder, compared to cases of common DLBCL (3 of 4 cases [75%] vs. 8 of 56 cases [14%]) [6]. The frequency of CD20-negative tumors among HIV-positive DLBCL cases is variable (2–26%) [7]. Nevertheless, to the best of our knowledge, there are no reported cases of EBV-positive B-cell lymphoma lacking a broad range of B-cell markers. McKelvie et al. reported a case of EBV-positive methotrexate-associated DLBCL that was negative for CD20 and CD79a [8]. Although that case and the present case were both positive for CD30 and MUM-1, McKelvie et al.’s case was positive for Pax-5 and ours was negative for Pax-5.

Our histological findings included the diffuse proliferation of CD20-negative and CD3-negative large cells including immunoblastic cells, plasmacytic cells, and multinuclear cells, which was compatible with various differential diagnoses: ALCL, extracavitary PEL, ALK-positive large B-cell lymphoma (ALK-LBCL), and PBL. In histology of our case CD30-positive HRS-like cells appeared, which mimicked ALCL and support our preliminary diagnosis before EBV testing by EBERs in situ hybridization. However, ALCL is exclusively EBV-negative and does not exhibit clonal rearrangement of the immunoglobulin genes. Extracavitary PEL usually develops in cases of systemic immunodeficiency, such as HIV infection, and is almost exclusively positive for human herpesvirus-8. ALK-LBCL is positive for ALK, CD138, and EMA, but is negative for EBV. Our initial IHC findings and the subsequent detection of EBV using EBERs in situ hybridization excluded ALCL, extracavitary PEL, and ALK-LBCL. PBL is positive for CD138 in almost all cases and also frequently expresses CD38 and MUM-1 [9, 10]. Although the results of IHC of our case could not clearly exclude PBL, diagnosis of PBL is unlikely because of lack of CD138 and CD38 expression. MUM-1 is one of markers for plasma cell differentiation, but previous studies showed that specificity of MUM-1 as a plasma cell marker is limited [11]. Montes-Moreno et al. categorized tumors with plasmablastic morphology and atypical immunophenotype (CD138 low or positive, CD20 low, Pax-5 low, MUM-1 positive, Blimp1 positive, and XBP1 negative) as PBL with variant (faulty) plasmablastic phenotype, and our case might be classified into this category according to their scheme [12]. Although we were initially unable to identify the lineage of tumor cells and histological type, the presence of necrotic foci and HRS-like cells in the tumor suggested an EBV-related disease, which prompted us to test for the expression of additional B-cell markers. Expression of B-cell-specific transcription factors detected by additional IHC and clonal rearrangement of the immunoglobulin genes confirmed our final diagnosis of EBV-positive DLBCL. Inadequate IHC using limited surface markers may lead to a misdiagnosis in cases of lymphomas with an unusual immunophenotype. It is important to miss a chance to perform further IHC in such cases, therefore detailed histological evaluation and accurate interpretation of results from a well-designed initial IHC panel are essential for reaching a correct diagnosis.

No previous studies have explained why B-cell markers are down-regulated in EBV-positive B-cell lymphomas, and the association of EBV infection with the suppression of B-cell markers remains unclear. Expression of latent EBV infection products influences the epigenetic status of the host cells [13], which might be associated with the regulation of B-cell marker expression. Down-regulation of CD20 in B-cell neoplasms is often related to plasmablastic features and terminal B-cell differentiation of the tumor cells [7]. Most CD20-negative B-cell lymphomas with plasmablastic features are positive for CD138, whereas tumor of the present case was negative for CD138. Therefore, plasmablastic features or plasmacytic differentiation is insufficient to explain the lack of these markers of our case. Moreover, the tumor did not express Pax-5, which is a critical B-cell lineage commitment factor and upregulates the expression of various B-cell differentiation markers [14]. Thus, it is possible that down-regulation of Pax-5 or aberrancy of other factors that modulate Pax-5 might have caused the loss of B-cell markers in our case. Mutations and translocations that involve the Pax-5 gene have been reported in some cases of B-cell lymphoma and leukemia [15–18]. In addition, the CD20-negative phenotype is observed in 26–27% of B-cell lymphomas after molecular-targeted therapy using rituximab [19, 20]. Several mechanisms can cause the down-regulation of CD20 after rituximab treatment: 1) mutational changes in the CD20 gene [21, 22], (2) aberrant transcriptional regulation of CD20 [23–25], (3) degradation of the CD20 protein by the ubiquitin-proteasome system [23], (4) other posttranscriptional or posttranslational changes in the regulation of CD20, and (5) genetic or transcriptional alterations in transcription factors that affect the expression of CD20 (e.g., PU.1 or Oct-2). Therefore, similar abnormalities involving Pax-5 or other common B-cell derivation factors may be responsible for the lack of B-cell markers in our case.

Previous studies have revealed that cases of DLBCL with reduced CD20 expression experience markedly inferior survival when they are treated using conventional CHOP (cyclophosphamide, hydroxydaunorubicin, Oncovin®, prednisolone) or rituximab-CHOP [26, 27]. Furthermore, a study of CD20-negative DLBCL cases, in which PBL and ALK-LBCL were carefully excluded, revealed that CD20-negative cases had a poorer response to conventional treatment and a poorer prognosis, compared to CD20-positive cases [7]. Moreover, CD20-negative cases had a higher proportion of the non-germinal center B-cell subtype, a higher proliferation index, and more frequent extranodal involvement, which might explain the biological aggressiveness of CD20-negative DLBCL [7].

In conclusion, we encountered an unusual case of EBV-positive DLBCL that was lacking various B-cell markers. This type of unusual phenotype often leads to an incorrect diagnosis, which can only be avoided by detailed evaluation of histopathology and appropriate utility of ancillary diagnostic tools, such as IHC for lineage-specific markers and gene rearrangement testing.

## Abbreviations

ALCL: Anaplastic large cell lymphoma
ALK: Anaplastic lymphoma kinase
ALK-LBCL: ALK-positive large B-cell lymphoma
CHOP: Cyclophosphamide, hydroxydaunorubicin, Oncovin®, prednisolone
DLBCL: Diffuse large B-cell lymphoma
EBER: EBV-encoded small RNA
EBV: Epstein-Barr virus
EMA: Epithelial membrane antigen
EPOCH: Etoposide, prednisolone, Oncovin®, cyclophosphamide, hydroxydaunorubicin
HE: Hematoxylin-eosin
HHV-8: Human herpesvirus-8
HIV: Human immunodeficiency virus
HRS: Hodgkin/Reed-Sternberg
IHC: Immunohistochemistry
PBL: plasmablastic lymphoma
PEL: Primary effusion lymphoma
THP-COP: 4’-O-tetrahydropyranyl adriamycin, cyclophosphamide, Oncovin®, prednisolone
